# Transcriptome Profile of a New Mouse Model of Spinocerebellar Ataxia Type 14 Implies Changes in Cerebellar Development

**DOI:** 10.3390/genes13081417

**Published:** 2022-08-09

**Authors:** Szilvia E. Mezey, Josef P. Kapfhammer, Etsuko Shimobayashi

**Affiliations:** Anatomical Institute, Department of Biomedicine, University of Basel, Pestalozzistrasse 20, 4056 Basel, Switzerland

**Keywords:** spinocerebellar ataxia, PKCγ, Purkinje cell

## Abstract

The autosomal dominant inherited spinocerebellar ataxias (SCAs) are a group of neurodegenerative disorders characterized by cerebellar atrophy and loss of Purkinje neurons. Spinocerebellar ataxia type 14 (SCA14) is a rare variant of SCAs caused by missense mutations or deletions in the *PRKCG* gene encoding the protein kinase C γ (PKCγ). Although mutated PKCγs are responsible for SCA14, it is still unclear exactly how mutated PKCγs are involved in SCA14 pathogenesis. Therefore, it is important to study how PKCγ signaling is altered in the cerebellum, which genes or signaling pathways are affected, and how this leads to neurological disease. In this study, we used a mouse line carrying a knock-in pseudo-substrate domain mutation in PKCγ (PKCγ-A24E) as an SCA14 model and performed RNA sequencing (RNA-seq) analysis at an early developmental timepoint (postnatal day 15) to investigate changes in the gene profile compared to wildtype mice. We analyzed both heterozygous (Het) PKCγ-A24E mice and homozygous (Homo) PKCγ-A24E mice for transcriptomic changes. The Het PKCγ-A24E mice reflects the situation observed in human SCA14 patient, while Homo PKCγ-A24E mice display stronger phenotypes with respect to Purkinje cell development and behavior. Our findings highlight an abundance of modifications affecting genes involved in developmental processes, suggesting that at least a part of the final phenotype is shaped by altered cerebellar development and is not only caused by changes in mature animals.

## 1. Introduction

Spinocerebellar ataxias (SCAs) are a heterogeneous group of dominantly inherited ataxias characterized by progressive cerebellar atrophy due mainly to Purkinje cell dysfunction and loss. Each type of SCA is caused by a mutation in a respective gene, with at least 48 SCA subtypes reported so far. Within SCAs, spinocerebellar ataxia type 14 (SCA14) (OMIM 605361) is an autosomal dominant neurodegenerative disease caused by mutations in the *PRKCG* gene encoding the protein kinase C γ (PKCγ) protein. The incidence of SCA14 is 1–4% of all autosomal dominant cerebellar ataxias [1,2], and patients suffer from a rather pure cerebellar ataxia, but the symptoms vary from patient to patient. PKCγ is a serine/threonine kinase predominantly expressed in the Purkinje cells of the cerebellum, serving as an important functional regulator for cell activities [3,4]. It is well known that dysfunction or dysregulation of Purkinje cells leads to cerebellar dysfunction and disease symptoms, since Purkinje cells are the only output neurons in the cerebellar cortex [5]. In the cerebellar cortex, PKCγ plays a crucial role for Purkinje cell development and function [6]. Previous studies have shown that increased PKC activity has a strong negative impact on Purkinje cell dendritic outgrowth [7]. Interestingly, an increased kinase activity of many SCA14 mutant PKCγs has been shown by in vitro assays, suggesting that a rise in kinase activity of mutated PKCγ leads to SCA14 [8]. The PKCγ-A24E mice carry a single point mutation located in the coding region of the pseudo-substrate domain of PKCγ (PKCγ-A24E), resulting in not only a constitutive activation of PKCγ but also its increased dephosphorylation and degradation, leading to a marked overall reduction of PKCγ protein expression. The Purkinje cells of PKCγ-A24E mice have stubby dendrites with reduced climbing fiber (CF) innervation, resulting in an impairment of motor behavior [9]. RNA profiling of tissue probes from the Purkinje cell layer and the molecular layer of 5 week old PKCγ-A24E mice indicated changes in heterozygous (Het) PKCγ-A24E mice in the expression of genes involved in mitochondrial oxidative metabolism. The affected pathways were involved in synaptogenesis and glutamate receptor signaling, as well as Purkinje cell development. Homozygous (Homo) PKCγ-A24E mice exhibited an altered regulation of ephrin receptors and ionotropic glutamate receptors [9]. The development of Purkinje cells and their innervation goes through a critical period in the first three postnatal weeks, when an overshoot of multiple CFs on each Purkinje cell are pruned back to achieve a 1:1 ratio of CFs and Purkinje cells by the end of the third postnatal week [10]. This pruning process is regulated by PKCγ, where an absence of PKCγ results in a failure to eliminate surplus CFs, leading to impaired motor coordination [11,12]. Along with its functional significance, the level of PKCγ also changes during postnatal development. Therefore, in the present study, we focused on changes in the transcriptome on postnatal day 15 (P15), when PKCγ is already present and the pruning process is ongoing, but the 1:1 ratio with CF is not yet established. The gene profile of PKCγ-A24E mice was analyzed using different approaches to achieve a comprehensive overview of changes in the transcriptome.

## 2. Materials and Methods

### 2.1. The SCA14 Mouse Model

Animal experiments were carried out in accordance with the EU Directive 2010/63/EU for animal experiments and were reviewed and permitted by Swiss authorities. The procedure of generating the PKCγ-A24E knock-in mouse line was described in [9]. The knock-in mutation was generated using the Cas9/CRISPR engineering system at the Center of Transgenic Models, University of Basel. The confirmed point mutation knock-in founders were crossed with FVB (Jackson Laboratory, Bar Harbor, ME, USA) mice to obtain wildtype (Wt), heterozygous (Het) PKCγ-A24E, and homozygous (Homo) PKCγ-A24E mice.

### 2.2. Total RNA Extraction and Total RNA Sequencing (RNA-Seq)

The cerebella from 12 mice aged P15 were isolated and harvested in RNAlater. Cerebellum lysates from four mice in each group were collected for total RNA isolation, following the instructions of the manufacturers of the Trizol and RNeasy Lipid Tissue Mini Kit (QIAGEN, Hilden, Germany). Samples were eluted in 15 μL of RNase-free H_2_O and quantified using a Nanodrop ND-1000 (Thermo Fisher Scientific, Inc., Waltham, MA, USA) spectrophotometer. RNA qualities were checked using Agarose Gel Electrophoresis and a Bioanalyzer 2100 (Agilent Technologies, Inc., Santa Clara, CA, USA). All samples were adjusted to 100 ng/μL and only used when the RNA integrity number (RIN) was ≥9.0. Library preparation and sequencing were performed with NextSeq 500 (Illumina, Inc., San Diego, CA, USA) at the Quantitative Genomics Facility of the University of Basel and the ETH Zurich at Basel.

### 2.3. RNA-Seq Data Collection and Analysis

Reads were aligned to the mouse genome (UCSC version mm10 analysis set) with STAR (version 2.7.0c) with default parameters except for allowing up to 10 hits to genome (outFilterMultimapNmax 10), reporting only one location for hits with equal score (outSAMmultNmax 1), and for filtering reads without evidence in spliced junction table (outFilterType “BySJout”). The output was sorted and indexed with samtools (v1.9). Stand-specific coverage tracks per sample were generated by tiling the genome in 20 bp windows and counting 5’ end of reads per window using the function bamCount from the Bioconductor package bamsignals (v1.22.0). These window counts were exported in bigWig format using the Bioconductor package rtracklayer (v1.50.0). Read and alignment quality was evaluated using the qQCReport function of the Bioconductor package QuasR (v1.30.0). The featureCounts function from Bioconductor package Rsubread (v2.4.3) was used to count the number of read (5’ ends) overlapping with the exons of each gene assuming an exon union model (with used gene model provided by ensembl v96).

The data were normalized by applying the TMM method from Bioconductor edgeR package (version 3.36.0). Lowly expressed genes were filtered out from further analysis using the filterByExpr function from edgeR package. The principal component analysis was based on 25% of most variable genes in the dataset. The differentially expressed genes were identified using the quasi-likelihood (QL) method implemented in edgeR package using sex as a covariate. Genes with FDR smaller than 0.05 were considered as differentially expressed. Gene set enrichment analysis was performed using the camera function from edgeR package for gene set collections provided in the MSigDb (v7.5.1) [13,14,15,16,17,18,19,20,21].

Cluster analysis of differentially expressed genes (DEGs) (FDR-corrected *p*-value < 0.05) was performed using the multiple protein analysis option of the String suite (string-db.org (accessed throughout 1 April–4 August 2022), version 11.5). Settings were adjusted to full String network type, where the edges indicate both functional and physical protein associations, with a required confidence score of 0.400 and an FDR-corrected *p*-value stringency of 0.05. The analysis used all available active interaction sources.

Gene expression analysis was carried out in R, using the Shiny app environment. The analysis comprised the mining of data for DEGs (FDR corrected *p*-value < 0.05). These DEGs were then subjected to principal component analysis and were filtered through a GSEA camera to light up relevant genes in the context of functional, positional, motif, or cell type gene sets.

Ingenuity Pathway Analysis (IPA) is a web-based biological analysis tool for omics data, and genomic data. We loaded RNA-seq data into QIAGEN’s IPA software (IPA^®^, QIAGEN, Redwood City, CA, USA, https://www.qiagenbioinformatics.com/products/ingenuity-pathway-analysis/ (accessed throughout 1 April–4 August 2022)) and performed a core analysis to highlight not only direct but also indirect relationships between genes and proteins. The IPA software shows the overlapping canonical pathways, upstream regulators, and affected gene/protein networks.

## 3. Results

### 3.1. Overview of Gene Expression Changes

**Figure 1:**
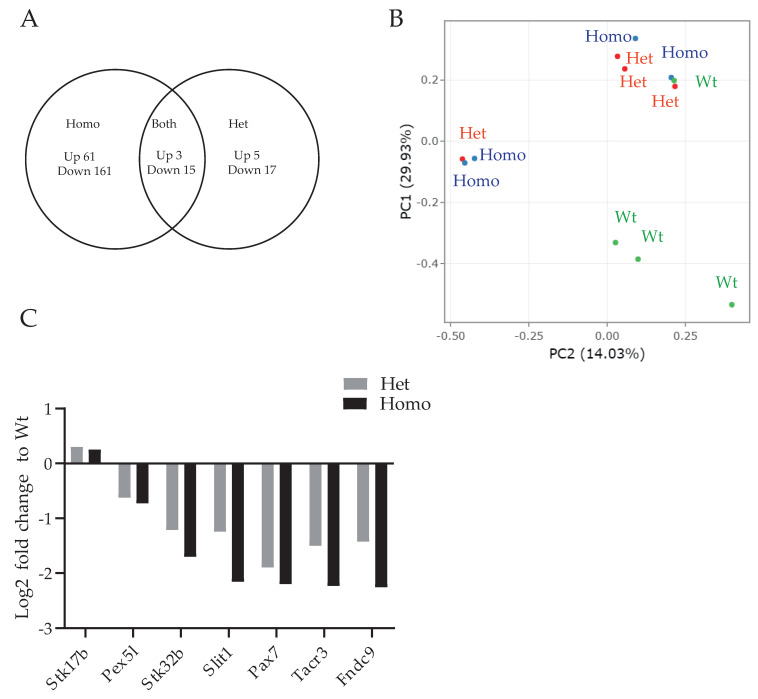
(**A**) Venn diagram of upregulated and downregulated genes in Het and Homo PKCγ-A24E mice. Out of 20,510 genes, 61 genes were upregulated, and 164 genes were downregulated in Homo PKCγ-A24E compared to Wt. Five genes were upregulated and 17 genes were downregulated in Het compared to Wt. (**B**) PCA analysis of representing individual animals. (**C**) Fold change of each significantly altered driving gene from PCA analysis.

We performed the RNA profiling of P15 Wt, Het, and Homo PKCγ-A24E mice (*n* = 4 per genotype). The cutoff was set at an FDR corrected *p*-value < 0.05 for both upregulated and downregulated genes. Altogether, 225 genes were differentially expressed in Homo, along with 22 genes in Het and 18 genes in both Homo and Het (Figure 1A and Table 1). For a list of significantly up- or downregulated genes in both Homo and Het PKCγ-A24E mice, see Supplementary Table S1. From the 18 DEGs shared by both Hetero and Homo PKCγ-A24E mice, three were significantly upregulated of which only *Stk17b* has been described in this context [22], and 15 genes were downregulated. PCA analysis indicated that both Het and Homo PKCγ-A24E mice showed a high individual variability (Figure 1B).

### 3.2. Highlighted Genes

The differential expression analysis genes highlighted some individual genes showing marked characteristics, identifying possible targets of mutant PKCγ for future studies of SCA14. Driving genes in the PCA analysis were defined as the genes that contributed the most to the separation of DEGs for the main sources of effects in Fold change (FC) expression. For PC1, the significantly altered driving genes were *Tacr3*, *Slit1*, *Stk32b*, and *Stk17b* in both Homo and Het PKCγ-A24E mice, as shown in Figure 1C.

The expression of *Tacr3* showed a significant reduction in both Homo and Het PKCγ-A24E mice, while, in some mice, it completely disappeared from the transcriptome. The *Tacr3* encoding the neurokinin 3 receptor is associated with G-proteins activating a phosphatidyl-inositol-calcium messenger system [23]. Since PKCγ is activated by calcium, changes in the expression level of *Tacr3* imply an adjustment of the calcium-regulating system in response to the A24E mutation in PKCγ.

**Figure 2:**
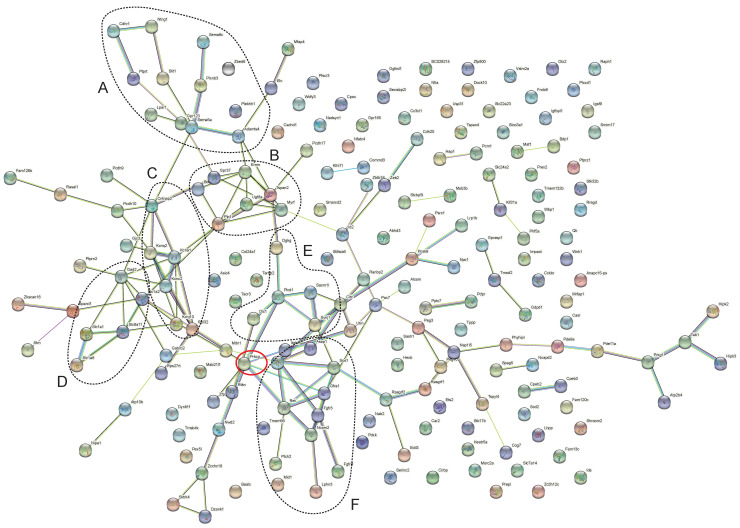
In the output of String analysis using the dataset derived from Homo PKCg-A24E, edges represent specific and meaningful protein–protein associations. Interaction types are coded by color. The analysis was carried out using PKCγ as an anchor point (red circle). DEGs were grouped into clusters on the basis of analyses of known and predicted protein–protein interactions (individual clusters enclosed by dotted lines). Individual gene names are visible after magnifying the image.

*Slit1* acts as a guidance cue in cellular migration, acting as a repulsive signal to prevent the growth of axons into specific areas [24], and this function is mediated by Robo1 receptors. The pruning of CFs on the dendritic tree of Purkinje cells requires an adjustment of signaling pathways related to the growth of axons. This group of genes were identified as DEGs in the cluster analysis as Group A (axon guidance, GO-MF GO:0007411); *Cdhr1*↓, *Ntng1*↓, *Ptprt*↓, *Slit1*↓, *Sema6c*↑, *Plxnb3*↓, *Lpar1*↓, *Sema5a*↓, *Adamts4*↓, *Otx2*↑, *Alcam*↓,↓: Downregulated; ↑: Upregulated(Figure 2). *Slit1* is also expressed in Purkinje cells and is involved in Purkinje cell dendritic development, as it is required for Purkinje cell dendrite self-avoidance [25].

*Stk32b* was significantly decreased in both Homo and Het PKCγ-A24E mice. *Stk32b* encodes the serine/threonine kinase YANK2, which has not been well characterized [26]. The only DEG significantly upregulated in both Homo and Het PKCγ-A24E mice, *Stk17b*, belongs to the same family of kinases and encodes the serine/threonine kinase DRAK2. This kinase was recently shown by our group to be a downstream effector of PKCγ and to regulate Purkinje cell dendritic development. Importantly, its expression is altered in diverse forms of spinocerebellar ataxia [22].

The paired box protein *Pax7*, peroxisomal biogenesis factor 5-like (*Pex5l*), and fibronectin type III-domain containing 9 (*Fndc9*) were significantly downregulated in both Homo and Het PKCγ-A24E mice. The functional role of these molecules in the cerebellum is still unclear, although *Pex5l* has been described to be specifically expressed by a distinctive group of cerebellar neurons [27].

### 3.3. Cluster Analysis

**Figure 3:**
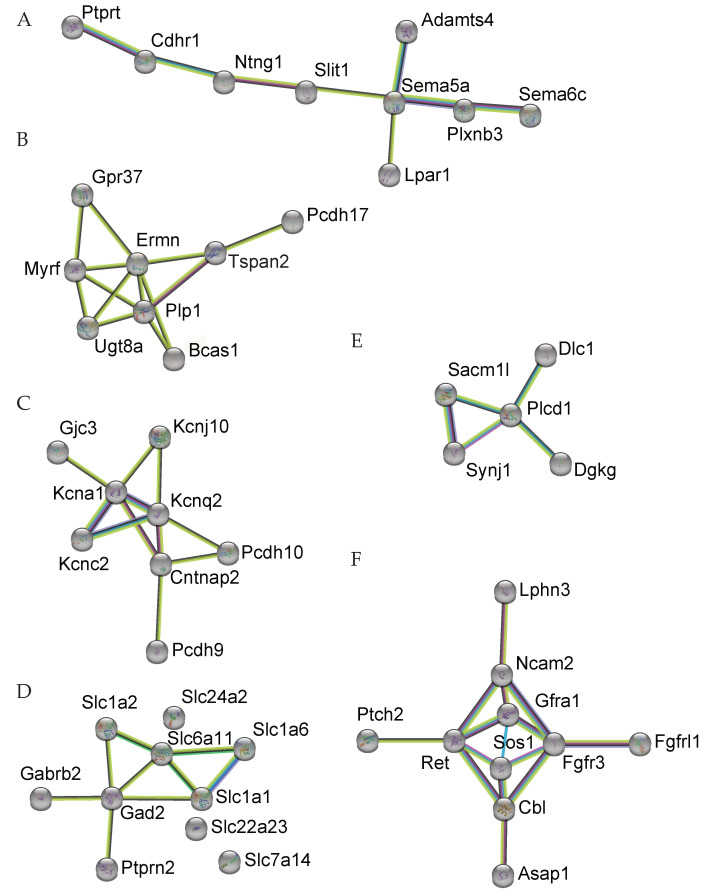
The cluster resulting from String analysis shown in Figure 2 was split into six groups on the basis of function. (**A**) Axon guidance genes (GO-MF GO:0007411). (**B**) Myelination-related genes (GO-BP GO:0042552). (**C**) Molecules related to voltage-gated potassium channels (GO-MF GO:0005249). (**D**) The superfamily of membrane solute carrier proteins (SLC). (**E**) The phosphatidylinositol signaling system (KEGG pathway mmu04070). (**F**) Molecules related to the transmembrane receptor protein tyrosine kinase signaling pathway (GO-BP:0007169).

Clusters of DEGs were identified on the basis of analyses of known and predicted protein–protein interactions using the String online analysis suite (Version 11.5). Groups of proteins were then summarized as GO or KEGG annotations. Figure 2 shows the overview of DEGs in Homo PKCγ-A24E mice. Regulated genes were tagged and connected by String to form a coherent cluster, where functionally associated genes were further categorized into groups (Figure 3).

Group A contains the axon guidance genes (GO-MF GO:0007411) affected in Homo: *Ntng1*↓, *Slit1*↓, *Sema5a*↓, *Sema6c*↑, *Plxnb3*↓, *Otx2*↑, *Alcam*↓, ↓: Downregulated; ↑: Upregulated (number of nodes: 9; number of edges: 8; average node degree: 1.78; average local clustering coefficient: 0.444; expected number of edges: 0; PPI enrichment *p*-value: 2.06 × 10^−12^). The best-known axon guidance molecules during the development of the central nervous system are the four main protein families Ephrins, Slits, Netrins, and Semaphorins, all of which are crucial for neural circuit establishment and synapse formation. Altered expression of these axon guidance genes results in abnormal synapse formation and leads to aberrant cerebellar circuitry [28].

Group B contains myelination related genes (GO-BP GO:0042552), all of which have a reduced expression: *Ermn*↓, *Gpr37*↓, *Tspan2*↓, *Tppp*↓, *Myrf*↓, *Ugt8a*↓, *Plp1*↓, *Bcas1*↓, *Fgfr3*↓, *Tspan2*↓, *Nab2*↓, *Plp1*↓, *Hexb*↓, *Kcnj10*↓, *Gjc3*↓ (number of nodes: 8; number of edges: 13; average node degree: 3.25; average local clustering coefficient: 0.738; expected number of edges: 0; PPI enrichment *p*-value = 1.0 × 10^−16^). This finding is compatible with a delayed maturation of the cerebellar circuitry in PKCγ-A24E mice due to a later onset of myelination after the completion of synapse formation.

Group C contains molecules related to voltage-gated potassium channels (GO-MF GO:0005249), one of which is found in both Homo and Het: *Kcna1*↓. The remainder are significantly regulated only in Homo: *Kcnq2*↓, *Kcnc2*↓, *Kcnj10*↓; *Cntnap2*↓ (number of nodes: 8; number of edges: 11; average node degree: 2.75; average local clustering coefficient: 0.754; expected number of edges: 0; PPI enrichment *p*-value = 5.55 × 10^−16^). *Kcna1* is associated with a rare neurological movement disorder known as episodic ataxia type 1 (EA1) [29]. In the cellular level, *Kcna1* is dominantly expressed in interneurons, including basket cells of the cerebellum, while *Kcnc2* is strongly expressed in Purkinje cells. The downregulation of these channels in the mutated mice also implies a developmental delay.

Group D contains the superfamily of membrane solute carrier genes (*Slc*); in Homo, only *Slc1a6*↑ (high affinity aspartate/glutamate transporter) becomes upregulated, whereas most others are downregulated: *Slc1a1*↓, *Slc1a2*↓, *Slc6a11*↓, *Slc7a14*↓, *Slc22a23*↓, *Slc24a2*↓, *Slco3a1*↓ (*Slc1a1* encodes neuronal/epithelial high affinity glutamate transporter, *Slc1a2* encodes glial high affinity glutamate transporter, *Slc6a1* encodes GABA neurotransmitter transporter, *Slc7a14* encodes cationic amino acid transporter, *Slc22a23* encodes organic ion transporter, *Slc24a2* encodes sodium/potassium/calcium exchanger, *Slco3a1* encodes organic anion transporter) (number of nodes: 10; number of edges: 9; average node degree: 1.8; average local clustering coefficient: 0.537; expected number of edges: 0; PPI enrichment *p*-value = 7.94 × 10^−13^). The SLC superfamily currently includes 458 transport proteins that transport a wide variety of substrates such as amino acids, inorganic ions, sugars, and neurotransmitters across plasma membranes or intracellular compartment membranes [30], and some SLCs are involved in the regulation of behavior [31] or are studied as drug targets [32]. Again, the predominant downregulation of these transporters is compatible with a developmental delay of the maturation of the cerebellar circuits in PKCγ-A24E mice.

The genes in Group E belong to the phosphatidylinositol signaling system (KEGG pathway mmu04070); *Dgkg*↓ (diacylglycerol kinase γ) encoding DGKγ was significantly downregulated in both Homo and Het, and is known to be associated with ataxia [33]. DGKγ is a protein directly associated with PKCγ and regulates its activation. Cluster E also contained *Dlc1*↓, *Plcd1*↑, *Sacm1l*↓, *Synj1*↓, all of which are connected to PI3 signaling. These changes are likely to be directly induced by the increased activity of PKCγ probably as a compensatory mechanism.

The genes in Group F are related to the transmembrane receptor protein tyrosine kinase signaling pathway (GO-BP:0007169): *Asap1*↓, *Cbl*↓, *Sos1*↓, *Gfra1*↓, *Ret*↓, *Fgfr3*↓, *Ptch2*↓, *Ncam2*↓, *Fgfrl1*↓, *Lphn3*↓ (number of nodes: 10; number of edges: 15; average node degree: 3; average local clustering coefficient: 0.66; expected number of edges: 0; PPI enrichment *p*-value = 5.87 × 10^−14^). As many of these genes are involved in cerebellar development, their predominant downregulation is also indicative of alterations and delays in the development and the maturation of cerebellar circuits.

### 3.4. Pathway Analysis

Ingenuity Pathway Analysis indicated that the following signaling pathways were significantly (FDR corrected *p*-value < 0.05) affected in Homo PKCγ-A24E using the top canonical pathways analysis: PTEN signaling: *Cbl*↓, *Fgfr3*↓, *Itgb8*↓, *Prex2*↓, *Sos1*↓, *Synj1*↓ (Table 2); G-protein-coupled receptor signaling: *Adgra1*↓, *Adgrl3*↓, *Casr*↓, *Cckbr*↓, *Gdpd1*↑, *Gpr37*↓, *Kcnq2*↓, *Lpar1*↓, *Nfatc4*↑, *Pde11a*↓, *Pde9a*↑, *Prex2*↓, *Sos1*↓, Tacr3↓ (Table 3); axonal guidance signaling: *Adamts4*↓, *Itgb8*↓, *Nfatc4*↑, *Ntng1*↓, *Plcd1*↑, *Plxnb3*↓, *Ptch2*↑, *Sema5a*↓, *Sema6c*↑, *Slit1*↓, *Sos1*↓ (Table 4); GABA receptor signaling: *Gabrb2*↓, *Gad2*↓, *Gpr37*↓, *Kcnq2*↓, *Slc6a11*↓ (Table 5).

Upstream analysis showed that *Sgk1* was one of the top hits in Homo PKCγ-A24E mice. *Sgk1* encodes the AGC kinase SGK1 and can be phosphorylated by the pleckstrin homology (PH) domain-containing protein PDK1, similar to PKCγ [34]. The target molecules of SGK1 are voltage-gated potassium channels (Group B in Figure 1) and solute carriers (Group D in Figure 1), supporting the hypothesis that SGK1 function might be altered in Homo mice. The expression and function of SGK1 is an interesting future target to be analyzed in PKCγ-A24E mice.

Furthermore, we looked into the molecules involved in diseases and biological functions, especially within the nervous system. The development of neural cells is significantly affected in Homo PKCγ-A24E mice (Supplementary Tables S2 and S3). Ten further ataxia-related DEGs of interest in Homo PKCγ-A24E mice are *Slc1a6*↑, *Synj1*↓, *Npc1*↓, *Slc1a1*↓, *Kcna1*↓, *Hexb*↓, *Slc1a2*↓, *Kcnj10*↓, *Ugt8a*↓, *Plp1*↓. Some of these genes were picked up by the cluster analysis suggesting that they may represent important molecules whose expression is affected by altered PKCγ kinase activity.

## 4. Discussion

In this study, we presented the gene clusters and networks showing an altered transcriptomic expression in the P15 PKCγ-A24E mouse characterized by an increased constitutive activity of PKCγ. We previously described the gene profile of the same SCA14 mouse model at a much later developmental timepoint [9], using RNA samples from 5 week old mice. The main aim of the current study was to investigate gene expression changes at an especially important timepoint of Purkinje cell dendritic development, where PKCγ plays a crucial role. Purkinje cells establish their elaborate dendritic tree postnatally in the human and rodent brain, and disruption of PKCγ function during Purkinje cell maturation results in reduced dendritic development, dysfunction of Purkinje cells, and motor deficits [7]. Activated PKCγ phosphorylates different target substrates, which in turn signal to inhibit dendritic development [35,36,37]. Of note, the reduction in Purkinje cell dendritic development at P15 is much more pronounced in Homo PKCγ-A24E compared to Het PKCγ-A24E mice [9]. From our RNA-seq analysis, 18 DEGs in both Homo and Het are listed in Supplementary Table S3, of which 15 genes have been characterized to date. Of these 15 DEGs, several are intriguing candidates related to the PKCγ signaling pathway and SCA pathology. For example, DGKγ is one of the molecules interacting directly with PKCγ. Deletion of DGKγ causes an impairment of motor coordination, long term depression and alterations in Purkinje cell dendritic development, due to increased activation of PKCγ [33]. Both DGKγ and PKCγ are strongly expressed in Purkinje cells; PKCγ directly phosphorylates DGKγ to regulate kinase activity [38]. It can be assumed that the increased activity of PKCγ with the A24E mutation results in more phosphorylated DGKγ, which in turn may alter transcriptomic expression. Further study of DGKγ and PKCγ regulation in PKCγ-A24E mice is needed to address this question in more detail. Another important molecule is *Stk17b*, which was previously upregulated in our different SCA14 mouse models and in SCA14 patients even though *Stk17b* is commonly downregulated in the cerebellum of SCA1, SCA2, and SCA7 mouse models [22,39]. *Stk17b* is the only gene among the 15 DEGs which was upregulated. However, in a different mouse model, the Moonwalker (Mwk), *Stk17b* is downregulated. Mwk is also a model for SCA caused by a gain-of-function mutation in the transient receptor potential (TRP) channel TRPC3 [40]. TRPC3 is phosphorylated by PKCγ and plays a pivotal role in Purkinje cell dendritic development and viability [41]. Stk17b emerges as one of the downstream molecules of the PKCγ–TRPC3 pathway regulating Purkinje cell morphology and functional activity.

*Sgk1* was not identified as a DEG but was tagged as an upstream molecule in the IPA analysis. The cluster analysis revealed several SLCs to be regulated in PKCγ-A24E with only *Slc1a6* being upregulated. *Slc1a6* encoding EAAT4 is a post-synaptic excitatory amino-acid transporter phosphorylated by SGK1 and is expressed abundantly in Purkinje cells [42]. SGK1 is regulated by mTORC2 [43] and was previously described to be significantly altered in the mTOR pathway in 5 week old PKCγ-A24E mice [9], thus marking it as a potential target of PKCγ.

Cluster analysis showed several voltage-gated potassium channels and SLCs being differentially expressed in PKCγ-A24E (Figure 3), some of which are strongly expressed in interneurons or glia cells. We observed that *Slc1a2* encoding EAAT2 located in the cerebellum (mainly in glial cells) was downregulated, EAAT4 (mainly located in Purkinje cells) was upregulated. Previous studies have shown that EAATs both from the pre- and the post-synaptic side could affect synaptic transmission, decreasing glutamate uptake and subsequently increasing the glutamate concentration in the synapse [44]. Interneuron activity, in turn, will be affected indirectly by altered Purkinje cell activity in the long term. Similar considerations apply to the voltage-gated potassium channels (Group C). *Kcna1*, which is strongly expressed in interneurons, was downregulated both in Homo and Het PKCγ-A24E mice. This suggests that the altered synaptic architecture in PKCγ-A24E mice leading to cerebellar dysfunction and neurodegeneration indirectly affects the expression of *Kcna1* in interneurons.

## 5. Conclusions

The present study revealed the gene clusters and signaling pathways altered in P15 PKCγ-A24E mice, an SCA14 mouse model with increased constitutive PKCγ activity. This cerebellar transcriptomic study revealed that using different analysis methods in combination is useful to better understand the gene profile and to find driving genes in the diseased brain. A limitation of this study is that we did not purify and isolate the Purkinje cells; therefore, the samples were heterogenous. In addition, due to the inherent heterogeneity of the PKCγ-A24E kinase activity itself, the differences in gene expression were small; thus, not all regulated genes may have been identified in this data. However, our results highlight several clusters of DEGs, many of which are linked to cerebellar development, as well as individual driving genes in both Homo and Her PKCγ-A24E mice. This study will stimulate further investigations on PKCγ signaling in Purkinje cells, particularly during postnatal development.

## Figures and Tables

**Table 1:** Differential gene expression detected with a false discovery rate (FDR)-corrected *p*-value < 0.05.

	Homo vs. WT	Hetero vs. WT	Homo vs. Hetero
Number of genes altogether	20,510	20,510	20,510
No significant change	20,285	20,488	20,510
Upregulated genes	61	5	0
Downregulated genes	164	17	0

**Table 2:** PTEN signaling-related genes in Homo PKCγ-A24E.

Genes	Log_2_FC	*p*-Value	FDR *p*-Value
*Cbl*	−0.219	0.0000233	0.0236
*Fgfr3*	−0.216	0.000263	0.0464
*Itgb8*	−0.36	0.000115	0.0382
*Prex2*	−0.276	0.0000319	0.0271
*Sos1*	−0.175	0.000346	0.0465
*Synj1*	−0.16	0.000403	0.0471

**Table 3:** G-protein-coupled receptor signaling genes in Homo PKCγ-A24E.

Genes	Log_2_FC	*p*-Value	FDR *p*-Value
*Adgra1*	−0.535	0.000494	0.0485
*Adgrl3*	−0.195	0.000158	0.0399
*Casr*	−0.904	0.0000363	0.0286
*Cckbr*	−1.398	0.000236	0.0461
*Gdpd1*	0.167	0.000519	0.0493
*Gpr37*	−0.56	0.000165	0.0399
*Kcnq2*	−0.23	0.0003	0.0464
*Lpar1*	−0.297	0.000438	0.0474
*Nfatc4*	0.481	0.000159	0.0399
*Pde11a*	−1.625	0.000314	0.0464
*Pde9a*	0.285	0.000242	0.0464
*Prex2*	−0.276	0.0000319	0.0271
*Sos1*	−0.175	0.000346	0.0465
*Tacr3*	−2.234	0.000526	0.0494

**Table 4:** Axonal guidance signaling genes in Homo PKCγ-A24E.

Genes	Log_2_FC	*p*-Value	FDR *p*-Value
*Adamts4*	−0.43	0.000202	0.0426
*Itgb8*	−0.36	0.000115	0.0382
*Nfatc4*	0.481	0.000159	0.0399
*Ntng1*	−0.499	0.00000993	0.0236
*Plcd1*	0.369	0.000128	0.0392
*Plxnb3*	−0.345	0.000445	0.0474
*Ptch2*	0.658	0.0000237	0.0236
*Sema5a*	−0.389	0.00000821	0.0236
*Sema6c*	0.273	0.000275	0.0464
*Slit1*	−2.16	0.000303	0.0464
*Sos1*	−0.175	0.000346	0.0465

**Table 5:** GABA receptor signaling genes in Homo PKCγ-A24E.

Genes	Log_2_FC	*p*-Value	FDR *p*-Value
*Gabrb2*	−0.324	0.000314	0.0464
*Gad2*	−0.301	0.000313	0.0464
*Gpr37*	−0.56	0.000165	0.0399
*Kcnq2*	−0.23	0.0003	0.0464
*Slc6a11*	−1.096	0.000134	0.0393

## Data Availability

The data presented in this study are available on request from the corresponding author.

## Supplementary Materials

The following supporting information can be downloaded at https://www.mdpi.com/article/10.3390/genes13081417/s1, Table S1: The genes significantly up- or downregulated in Het PKCγ-A24E mice; Table S2: The genes significantly up- or downregulated in Homo PKCγ-A24E mice; Table S3: The genes significantly regulated in Homo PKCγ-A24E mice related to developmental neural cells with IPA analysis.
