# Supplementary material for: Transcriptome Profile of a New Mouse Model of Spinocerebellar Ataxia Type 14 Implies Changes in Cerebellar Development
Source: Genes (Basel). 2022 Aug 9;13(8):1417. doi: 10.3390/genes13081417 (PMC9407720; doi:10.3390/genes13081417)
Supplement: Supplementary file 1 [file genes-13-01417-s001.zip › genes-1829986-supplementary.pdf]

Supplementary Tables

Supplementary Table S1. The genes significantly up- or downregulated in Het PKCγ-A24E mice. Bold highlighted genes are differentially expressed in both Homo and Hetero PKCg-A24E mice.

Genes	log2FC	p-value	FDR	Regulation
**Stk17b**	0.3006	3.81E-05	0.04781	UP
Mrfap1	0.2246	5.13E-05	0.04781	UP
**Wdfy3**	-0.2266	3.11E-05	0.04781	DOWN
**Ranbp2**	-0.2276	3.57E-05	0.04781	DOWN
**Bdp1**	-0.2777	4.90E-06	0.03873	DOWN
**Prex2**	-0.2839	4.85E-05	0.04781	DOWN
**Kcna1**	-0.304	8.91E-06	0.03873	DOWN
**Fam126b**	-0.3262	2.61E-05	0.04781	DOWN
**Zbed6**	-0.349	1.32E-05	0.03873	DOWN
**Hipk2**	-0.351	3.80E-05	0.04781	DOWN
**Cpeb3**	-0.3512	3.25E-06	0.03873	DOWN
**Stxbp5l**	-0.4039	1.75E-05	0.03991	DOWN
**Dgkg**	-0.4091	5.11E-05	0.04781	DOWN
**Ntng1**	-0.4608	4.47E-05	0.04781	DOWN
**Ncam2**	-0.5396	5.13E-05	0.04781	DOWN
**Mid1-ps1**	-2.6683	1.04E-05	0.03873	DOWN

Supplementary Table S2. The genes significantly up- or downregulated in Homo PKCγ-A24E mice. Bold highlighted genes are differentially expressed in both Homo and Hetero PKCg-A24E mice.

**Genes**	log2FC	P.Value	FDR	Regulation
Psrc1	1.1505	1.75E-04	0.04000	UP
Mfap4	0.9363	3.81E-04	0.04689	UP
Anapc15-ps	0.7787	4.11E-04	0.04715	UP
Pcsk9	0.7576	2.31E-04	0.04563	UP
Igfbpl1	0.7405	4.17E-04	0.04744	UP
Igsf9	0.7026	3.31E-04	0.04640	UP
Ptch2	0.6584	2.37E-05	0.02360	UP
Eln	0.4881	4.04E-06	0.01884	UP
Nfatc4	0.4807	1.59E-04	0.03986	UP
Serinc2	0.4542	1.80E-05	0.02360	UP
Plcd1	0.3695	1.28E-04	0.03921	UP
Nadsyn1	0.3471	5.06E-04	0.04876	UP
Tmem265	0.3341	4.73E-04	0.04744	UP
Slc1a6	0.3273	2.55E-04	0.04640	UP
Dynlt1f	0.3252	2.95E-04	0.04640	UP
Lhpp	0.3181	1.49E-04	0.03986	UP
Cirbp	0.3160	8.06E-05	0.03612	UP
Mir9-3hg	0.2947	3.78E-04	0.04689	UP

Tarbp2	0.2883	2.02E-05	0.02360	UP
Pde9a	0.2850	2.42E-04	0.04638	UP
Otx2	0.2796	1.06E-04	0.03818	UP
Sema6c	0.2733	2.75E-04	0.04640	UP
Phf5a	0.2630	3.00E-04	0.04640	UP
Cog7	0.2613	3.97E-04	0.04689	UP
Mab21l1	0.2595	8.44E-05	0.03612	UP
Spag5	0.2580	3.63E-04	0.04689	UP
Plscr3	0.2560	1.24E-04	0.03852	UP
**Stk17b**	0.2547	1.21E-04	0.03852	UP
Tmsb4x	0.2419	3.94E-04	0.04689	UP
Maf1	0.2379	5.51E-05	0.03612	UP
Ncapd2	0.2339	5.37E-04	0.04942	UP
Zbtb18	0.2325	2.64E-04	0.04640	UP
Id2	0.2318	3.00E-05	0.02711	UP
Wbp1	0.2226	1.34E-04	0.03934	UP
Paxx	0.2163	3.98E-04	0.04689	UP
Nfia	0.2161	4.11E-04	0.04715	UP
Rps27rt	0.2121	3.68E-04	0.04689	UP
Tab1	0.2112	1.08E-04	0.03818	UP
Rpl32	0.2028	3.24E-04	0.04640	UP
Smarcd2	0.2008	3.36E-04	0.04640	UP
Commd3	0.1969	4.36E-04	0.04744	UP
Tspan4	0.1947	4.64E-04	0.04744	UP
Tpt1-ps3	0.1890	2.58E-04	0.04640	UP
Morc2a	0.1704	4.45E-04	0.04744	UP
Gdpd1	0.1674	5.19E-04	0.04930	UP
Ids	-0.1477	4.39E-04	0.04744	DOWN
Synj1	-0.1603	4.03E-04	0.04715	DOWN
Tspyl4	-0.1608	1.90E-04	0.04177	DOWN
Hipk3	-0.1641	3.68E-04	0.04689	DOWN
**Wdfy3**	-0.1694	3.01E-04	0.04640	DOWN
Sos1	-0.1746	3.46E-04	0.04651	DOWN
Cltc	-0.1784	1.62E-04	0.03986	DOWN
**Bdp1**	-0.1785	2.67E-04	0.04640	DOWN
Pcm1	-0.1792	1.98E-04	0.04221	DOWN
Kif21a	-0.1814	4.44E-04	0.04744	DOWN
Ptprz1	-0.1823	4.80E-04	0.04758	DOWN
Wnk1	-0.1825	2.28E-04	0.04536	DOWN
Npc1	-0.1839	3.34E-04	0.04640	DOWN
Utrn	-0.1866	7.49E-05	0.03612	DOWN
Gprasp1	-0.1868	4.48E-04	0.04744	DOWN
Zcchc18	-0.1881	5.47E-04	0.04985	DOWN
Adgrl3	-0.1945	1.58E-04	0.03986	DOWN
**Ranbp2**	-0.1954	8.47E-05	0.03612	DOWN
Raph1	-0.1999	1.38E-04	0.03986	DOWN

Fam120c	-0.2009	5.46E-04	0.04985	DOWN
Tmod2	-0.2034	3.42E-04	0.04651	DOWN
Heatr5a	-0.2070	3.23E-04	0.04640	DOWN
Strn	-0.2078	1.14E-04	0.03818	DOWN
Sacm1l	-0.2111	3.86E-04	0.04689	DOWN
Sparcl1	-0.2135	3.97E-04	0.04689	DOWN
Secisbp2l	-0.2136	6.61E-05	0.03612	DOWN
Fgfr3	-0.2164	2.63E-04	0.04640	DOWN
Dlc1	-0.2167	2.88E-04	0.04640	DOWN
Pdpr	-0.2171	3.76E-04	0.04689	DOWN
Slco3a1	-0.2176	3.27E-04	0.04640	DOWN
Usp31	-0.2187	1.58E-04	0.03986	DOWN
Cbl	-0.2194	2.33E-05	0.02360	DOWN
Ret	-0.2195	4.10E-04	0.04715	DOWN
Phyhipl	-0.2217	2.19E-04	0.04401	DOWN
Rragd	-0.2273	4.35E-04	0.04744	DOWN
Qk	-0.2280	1.63E-04	0.03986	DOWN
Kcnq2	-0.2297	3.00E-04	0.04640	DOWN
Pptc7	-0.2317	3.48E-04	0.04651	DOWN
Mob3b	-0.2325	5.11E-04	0.04876	DOWN
Cpeb2	-0.2376	9.72E-05	0.03787	DOWN
Prepl	-0.2377	2.12E-04	0.04335	DOWN
Zeb2	-0.2385	4.70E-04	0.04744	DOWN
Slc1a1	-0.2391	4.65E-04	0.04744	DOWN
Mdn1	-0.2402	1.46E-04	0.03986	DOWN
Fam13c	-0.2409	9.24E-05	0.03716	DOWN
Pdxk	-0.2446	2.09E-05	0.02360	DOWN
Tmem56	-0.2454	8.84E-06	0.02360	DOWN
Cntnap2	-0.2510	2.39E-04	0.04633	DOWN
**Hipk2**	-0.2579	4.37E-04	0.04744	DOWN
Lrp1b	-0.2645	2.68E-04	0.04640	DOWN
Ets2	-0.2652	1.21E-04	0.03852	DOWN
**Fam126b**	-0.2693	9.07E-05	0.03716	DOWN
Peg3	-0.2711	6.90E-05	0.03612	DOWN
Sash1	-0.2712	4.60E-04	0.04744	DOWN
Pcdh9	-0.2725	1.84E-05	0.02360	DOWN
Zfp800	-0.2742	3.49E-04	0.04651	DOWN
**Prex2**	-0.2758	3.19E-05	0.02711	DOWN
Baalc	-0.2803	4.68E-04	0.04744	DOWN
Slc22a23	-0.2856	5.36E-04	0.04942	DOWN
Slitrk4	-0.2891	1.67E-04	0.03986	DOWN
Atp2b4	-0.2902	2.10E-04	0.04335	DOWN
**Zbed6**	-0.2917	4.71E-05	0.03452	DOWN
Ptprt	-0.2932	3.91E-04	0.04689	DOWN
Lpar1	-0.2965	4.38E-04	0.04744	DOWN
Ogfod1	-0.2974	2.91E-04	0.04640	DOWN

Mir5114	-0.2985	4.40E-04	0.04744	DOWN
Gad2	-0.3008	3.13E-04	0.04640	DOWN
Slc7a14	-0.3018	8.54E-05	0.03612	DOWN
**Kcna1**	-0.3045	4.19E-06	0.01884	DOWN
Hexb	-0.3072	4.80E-04	0.04758	DOWN
Abhd3	-0.3089	1.96E-04	0.04221	DOWN
Slc24a2	-0.3156	7.41E-05	0.03612	DOWN
Cpox	-0.3170	1.05E-04	0.03818	DOWN
**Dgkg**	-0.3209	3.19E-04	0.04640	DOWN
**Stxbp5l**	-0.3210	9.97E-05	0.03787	DOWN
Gabrb2	-0.3242	3.14E-04	0.04640	DOWN
**Cpeb3**	-0.3267	3.71E-06	0.01884	DOWN
Prkg1	-0.3269	3.70E-04	0.04689	DOWN
Slc1a2	-0.3273	3.04E-05	0.02711	DOWN
Bcas1	-0.3333	4.49E-04	0.04744	DOWN
Asap1	-0.3335	3.08E-04	0.04640	DOWN
Ptprn2	-0.3340	8.10E-05	0.03612	DOWN
Edil3	-0.3344	2.87E-04	0.04640	DOWN
Tspan2	-0.3384	2.74E-04	0.04640	DOWN
Plxnb3	-0.3447	4.45E-04	0.04744	DOWN
Zc3h12c	-0.3455	5.30E-04	0.04938	DOWN
Dock10	-0.3516	3.30E-05	0.02711	DOWN
Nipa1	-0.3553	3.72E-04	0.04689	DOWN
Car2	-0.3574	1.66E-04	0.03986	DOWN
Itgb8	-0.3599	1.15E-04	0.03818	DOWN
Dzank1	-0.3682	2.71E-04	0.04640	DOWN
Alcam	-0.3715	2.51E-04	0.04640	DOWN
Cdh20	-0.3822	1.02E-04	0.03808	DOWN
Rtkn	-0.3873	2.88E-04	0.04640	DOWN
Tppp	-0.3875	1.07E-05	0.02360	DOWN
Sema5a	-0.3891	8.21E-06	0.02360	DOWN
Klhl11	-0.3964	1.83E-04	0.04079	DOWN
Kcnj10	-0.4117	4.85E-04	0.04780	DOWN
Myrf	-0.4187	1.63E-05	0.02360	DOWN
Gjc3	-0.4236	5.06E-04	0.04876	DOWN
Adamts4	-0.4298	2.02E-04	0.04263	DOWN
Nap1l5	-0.4403	1.91E-04	0.04177	DOWN
Cachd1	-0.4499	1.77E-04	0.04000	DOWN
Nwd2	-0.4529	4.41E-05	0.03348	DOWN
Impact	-0.4788	1.31E-04	0.03934	DOWN
Nab2	-0.4795	3.96E-04	0.04689	DOWN
Pcdh17	-0.4825	2.15E-05	0.02360	DOWN
Pcdh10	-0.4826	1.13E-04	0.03818	DOWN
Rasal1	-0.4869	2.12E-05	0.02360	DOWN
**Ntng1**	-0.4990	9.93E-06	0.02360	DOWN
Zfp365	-0.5023	3.35E-04	0.04640	DOWN

Plekhh1	-0.5201	1.75E-04	0.04000	DOWN
Sox2ot	-0.5278	1.15E-04	0.03818	DOWN
Fgfrl1	-0.5284	3.77E-04	0.04689	DOWN
Adgra1	-0.5350	4.94E-04	0.04849	DOWN
Cx3cl1	-0.5381	3.43E-04	0.04651	DOWN
Ugt8a	-0.5472	7.33E-05	0.03612	DOWN
Atp10b	-0.5544	1.60E-04	0.03986	DOWN
Gpr37	-0.5603	1.65E-04	0.03986	DOWN
Ermn	-0.5673	8.63E-05	0.03612	DOWN
Plp1	-0.5684	4.31E-04	0.04744	DOWN
Shroom2	-0.5714	5.37E-04	0.04942	DOWN
Gfra1	-0.6082	2.60E-04	0.04640	DOWN
Rasgrf1	-0.6121	1.78E-05	0.02360	DOWN
**Ncam2**	-0.6251	4.59E-06	0.01884	DOWN
Hap1	-0.6305	4.05E-04	0.04715	DOWN
Pabpc1l2b-ps	-0.6715	1.23E-04	0.03852	DOWN
Tmem132b	-0.6859	4.74E-04	0.04744	DOWN
Peg10	-0.7091	4.68E-04	0.04744	DOWN
Pex5l	-0.7278	1.60E-04	0.03986	DOWN
Plcxd1	-0.7387	3.30E-04	0.04640	DOWN
Vstm2a	-0.7398	3.11E-04	0.04640	DOWN
Casr	-0.9043	3.63E-05	0.02861	DOWN
Gpr165	-0.9428	3.19E-04	0.04640	DOWN
St8sia6	-0.9644	2.13E-04	0.04335	DOWN
Slc6a11	-1.0963	1.34E-04	0.03934	DOWN
Smim17	-1.1013	7.32E-05	0.03612	DOWN
Zkscan16	-1.1840	5.54E-05	0.03612	DOWN
Col24a1	-1.1873	3.37E-04	0.04640	DOWN
Mid1	-1.3664	8.34E-05	0.03612	DOWN
Cckbr	-1.3983	2.36E-04	0.04609	DOWN
Cdhr1	-1.4899	5.11E-04	0.04876	DOWN
Pde11a	-1.6250	3.14E-04	0.04640	DOWN
Kcnc2	-1.6576	1.49E-04	0.03986	DOWN
Stk32b	-1.7024	3.78E-04	0.04689	DOWN
Rasgrf2	-1.8574	1.76E-04	0.04000	DOWN
Asic4	-2.0090	1.48E-04	0.03986	DOWN
**Mid1-ps1**	-2.1407	5.39E-05	0.03612	DOWN
Slit1	-2.1595	3.03E-04	0.04640	DOWN
Pax7	-2.2033	8.07E-05	0.03612	DOWN
Tacr3	-2.2342	5.26E-04	0.04938	DOWN
Fndc9	-2.2579	2.09E-04	0.04335	DOWN

Supplementary Table S3. The genes significantly regulated in Homo PKCγ-A24E mice related to developmental neural cells with IPA analysis.

Genes	FDR p-value
Kcnj10	0.000485
Ptprz1	0.00048
Zeb2	0.00047
Hipk2	0.000437
Plp1	0.000431
Nfia	0.000411
Ret	0.00041
Sparcl1	0.000397
Prkg1	0.00037
Cx3cl1	0.000343
Znf365	0.000335
Igsf9	0.000331
Dgkg	0.000319
Gabrb2	0.000314
Slit1	0.000303
Tspan2	0.000274
Zbtb18	0.000264
Fgfr3	0.000263
Alcam	0.000251
Cntnap2	0.000239
Adamts4	0.000202
Slitrk4	0.000167
Gpr37	0.000165
Nfatc4	0.000159
Adgrl3	0.000158
Raph1	0.000138
Otx2	0.000106
Mid1	0.0000834
Utrn	0.0000749
Ugt8	0.0000733
Dock10	0.000033
Prex2	0.0000319
Id2	0.00003
Rasgrf1	0.0000178
Myrf	0.0000163
Tppp	0.0000107
Ntng1	0.00000993
Ncam2	0.00000459
Cpeb3	0.00000371
